# First analyses of lysine succinylation proteome and overlap between succinylation and acetylation in *Solenopsis invicta* Buren (Hymenoptera: Formicidae)

**DOI:** 10.1186/s12864-021-08285-8

**Published:** 2022-01-17

**Authors:** Jingwen Ye, Jun Li

**Affiliations:** Guangdong Key Laboratory of Animal Conservation and Resource Utilization, Guangdong Public Laboratory of Wild Animal Conservation and Utilization, Institute of zoology, Guangdong Academy of Sciences, Xingang West Road 105, Guangzhou, Guangdong Province 510260 People’s Republic of China

**Keywords:** Succinylation, Acetylation, Proteome, *Solenopsis invicta*, Overlap

## Abstract

**Background:**

Lysine succinylation (Ksu) exists in both eukaryotes and prokaryotes, and influences a variety of metabolism processes. However, little attention has been paid to Ksu in insects, especially the notorious invasive pest *Solenopsis invicta*.

**Results:**

In this study, the first analyses of Ksu proteome and overlap between Ksu and lysine acetylation (Kac) in *S*. *invicta* were presented. 3753 succinylated sites in 893 succinylated proteins were tested. The dihydrolipoyl dehydrogenase, V-type proton ATPase subunit G, and tubulin alpha chain all had evolutionary conservatism among diverse ant or bee species. Immunoblotting validation showed that there were many Ksu protein bands with a wide range of molecular mass. In addition, 1230 sites in 439 proteins were highly overlapped between Ksu and Kac. 54.05% of Ksu proteins in cytoplasm were acetylated. The results demonstrated that Ksu may play a vital part in the allergization, redox metabolism, sugar, fat, and protein metabolism, energy production, immune response, and biosynthesis of various secondary metabolites.

**Conclusions:**

Ksu and Kac were two ubiquitous protein post-translational modifications participated in a variety of biological processes. Our results may supply rich resources and a starting point for the molecular basic research of regulation on metabolic pathways and other biological processes by succinylation and acetylation.

**Supplementary Information:**

The online version contains supplementary material available at 10.1186/s12864-021-08285-8.

## Background

Lysine residues can be modified by a large number of post-translational protein acylation modifications, namely, succinylation, acetylation, β-hydroxybutyrylation, 2-hydroxyisobutyrylation, and biotinylation [1]. Lysine succinylation (Ksu) transforms the side chains of cationic lysine into anions, and has important potential efforts on the structure and function of proteins [2]. Ksu is a novel discovered reversible post-translational modification (PTM), which exists in both eukaryotes and prokaryotes and influences a variety of metabolism processes [1]. Ksu may functionalize proteins mainly by non-enzymatic mechanisms [3]. Ksu couples the cyclic metabolism of tricarboxylic acid (TCA) with the changes of activities, structures, and charges of proteins participated in multiple cellular processes through succinyl-CoA [4]. Comprehensive study of proteome has shown that Ksu strides over various biological compartments, but mainly centralizes on mitochondria [5].

Protein sucinylome, whose aim was to determine the comprehensive Ksu sites at the proteomic level, was carried out in plants, microorganisms, and animals [6]. The comprehensive succinylome was first reported in the traditional Chinese medicine herb *Pogostemon cablin* (Blanco) Benth, which broadened the range of Ksu in plants [7]. Gao et al. [8] utilized mass spectrometry-based proteomics to enrich Ksu peptides by immunoprecipitation, and conducted a comprehensive localization of Ksu in zebrafish *Danio rerio*. The global view of succinate group in the rice blast disease *Pyricularia oryzae* for the first time was supplied, which might contribute to seek latent pathogenicity-related proteins to control *P. oryzae* [9]. The proteomes of Ksu and Kac were analyzed, and these two modifications were widely involved in the metabolism of the seed embryos of germinating rice *Oryza sativa* [6]. Zhen et al. [10] firstly analyzed the proteomes of Ksu and Kac in the seedling leaves of *Brachypodium distachyon* L, and showed that Ksu and Kac could be used as switches to control the activities of some key enzymes and ensure the proper developmental function of *B. distachyon* accession 21. The first succinyl and acetyl groups of *Pseudomonas aeruginosa* PA14, which were cultured at the exits of four diverse carbon sources, were presented [11].

*Solenopsis invicta* is an invasive pest of notorious reputation in the world. Because *S. invicta* spreads rapidly, it has led to many ecological problems and huge economic losses to many countries, prompting researchers to conduct in-depth study on its invasion causes [12]. The sting of *S. invicta* can lead to skin redness, swelling, anaphylactic shock, and even death [13]. *S. invicta* depends on quantitative and behavioral advantages to take over native species, and has a great impact on resident ants after establishment [14]. The acetylome in *S. invicta* for the first time revealed that extensive functions were modified by Kac [15]. However, little attention has been paid to Ksu in insects. In this study, the first analyses of Ksu proteome and overlap between Ksu and Kac in this notorious invasive species *S*. *invicta* were presented.

## Results

### Proteomic profiling of Ksu in *S. invicta*

**Fig. 1:**
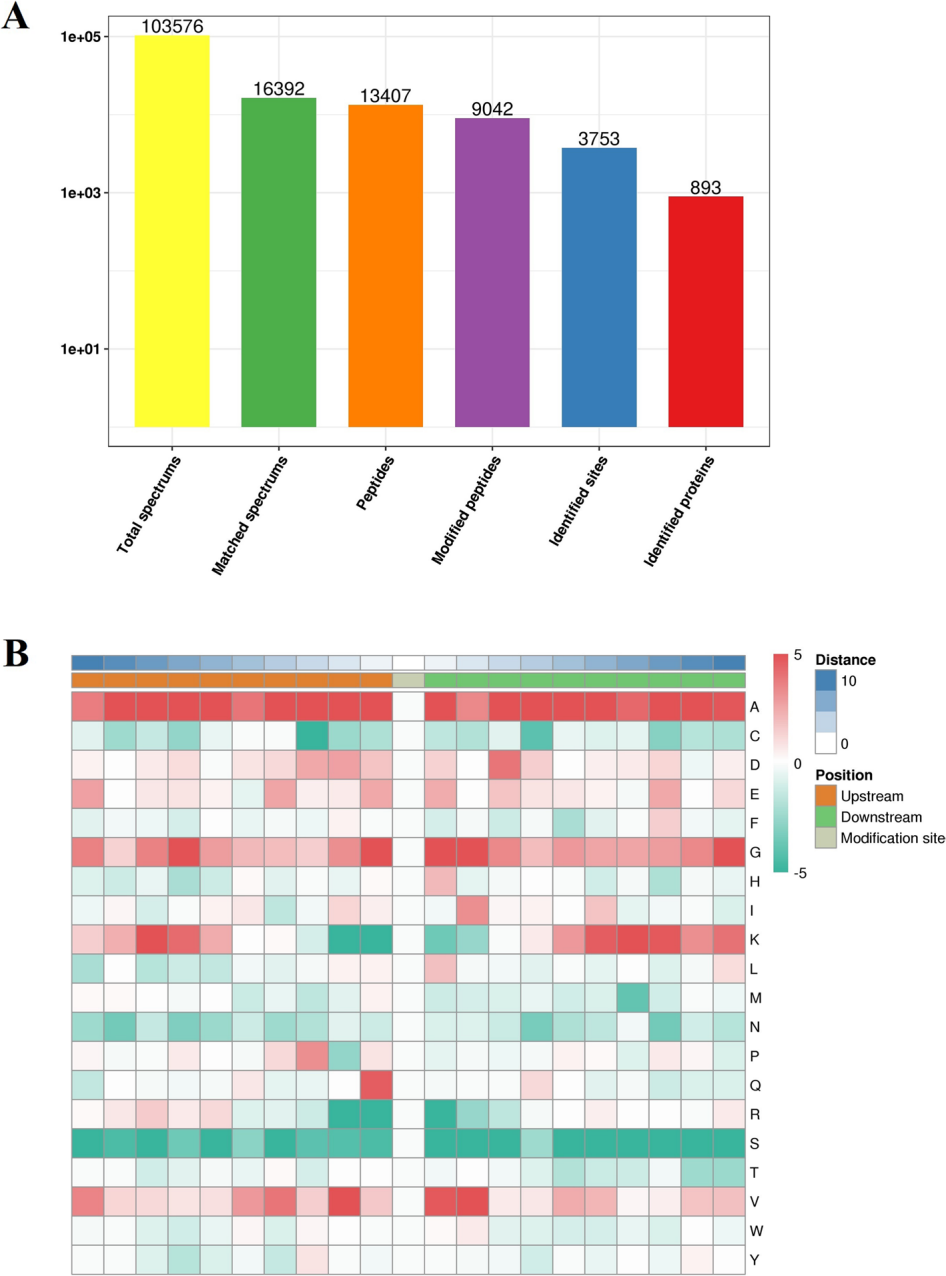
Identification of lysine succinylation (Ksu) sites in *Solenopsis invicta*. **A** Basic statistical results of MS. **B** Heat map analysis of motif enrichment of amino acids around Ksu sites. Red denotes significantly enriched amino acids near the Ksu site, while green denotes significantly reduced amino acids near the Ksu site

The length of all Ksu peptides ranged from 7 to 37 amino acids, and most of them ranged from 7 to 19 amino acids (S1 Fig. A-C). 3753 non-redundant succinylated sites in 893 non-redundant succinylated proteins were tested (Fig. 1A). There were 306 proteins (40.89%) that included one Ksu site, and there were 179 proteins (23.90%) that included five or more Ksu sites. 30.57 proteins (4.10%) had more than 15 Ksu sites. There were 4.04 sites per protein (S2 Fig. A-C). 1355 sites were discovered to contain amino acid sequence around − 10 to + 10 positions of succinylated lysine. Five conserved motifs were significantly overrepresented around the Ksu sites, such as K_(su)_ xxxxxxK, AK_(su)_, GK_(su)_, K_(su)_ A, and QK_(su)_. “x” meant a random amino acid residue. Alkaline amino acid (K) and hydrophobic amino acid (A) were located in the upstream of Ksu sites, while hydrophobic amino acid (A) and hydrophilic amino acids (G and Q) were located in the downstream of Ksu sites. K_(su)_ xxxxxxK was the most abundant, which frequently occurred at the + 7 position (Fig. 1B).

### Gene ontology (GO) classification and subcellular distribution of succinylated proteins

**Fig. 2:**
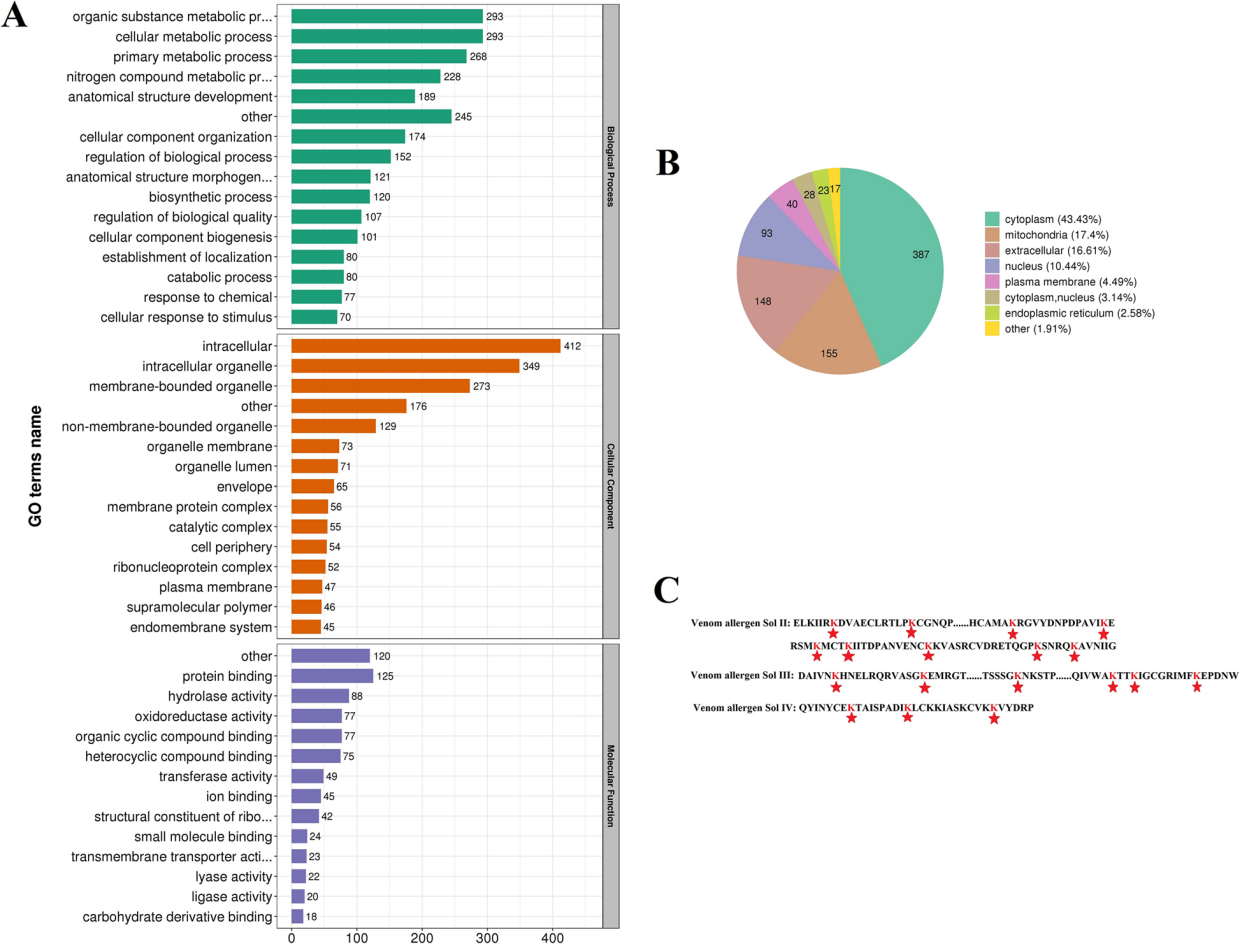
Analyses of GO classification and subcellular distribution of the identified lysine succinylation (Ksu) proteins in *Solenopsis invicta*. **A** GO classification analysis in biological process, cellular component, and molecular function. **B** Subcellular localization. **C** Ksu sites of the venom allergens Sol i II, Sol i III, and Sol i IV seated in the extracellular. The red stars indicated conserved lysine succinylated residues

In order to understand the possible roles of Ksu, GO classification of the identified proteins was conducted. The analysis of biological process showed that the main functions of Ksu proteins were involved in the cellular metabolic process, organic substance metabolic process, primary metabolic process, and nitrogen compound metabolic process. Within the category of cellular component, the Ksu proteins were mostly localized in the intracellular, intracellular organelle, membrane-bounded organelle, and non-membrane-bounded organelle. With respect to molecular functions, the proteins were predominantly involved in protein binding, hydrolase activity, oxidoreductase activity, and organic cyclic compound binding (Fig. 2A).

43.43% of the Ksu proteins occurred in the cytoplasm, 17.40% of them occurred in the mitochondria, 16.61% of them were located in the extracellular, and 10.44% of them were located in the nucleus (Fig. 2B). It was found that the venom allergens Sol i II (10 Ksu sites), Sol i III (6 Ksu sites), and Sol i IV (10 Ksu sites) were localized in the extracellular (Fig. 2C). The MS^2^ spectra of the amino acid position K29 and K40 in Sol i II, K71 and K204 in Sol i III, and K74 and K91 in Sol i IV were shown in S3 Fig. A-F.

### GO, KEGG, and domain enrichments

**Fig. 3:**
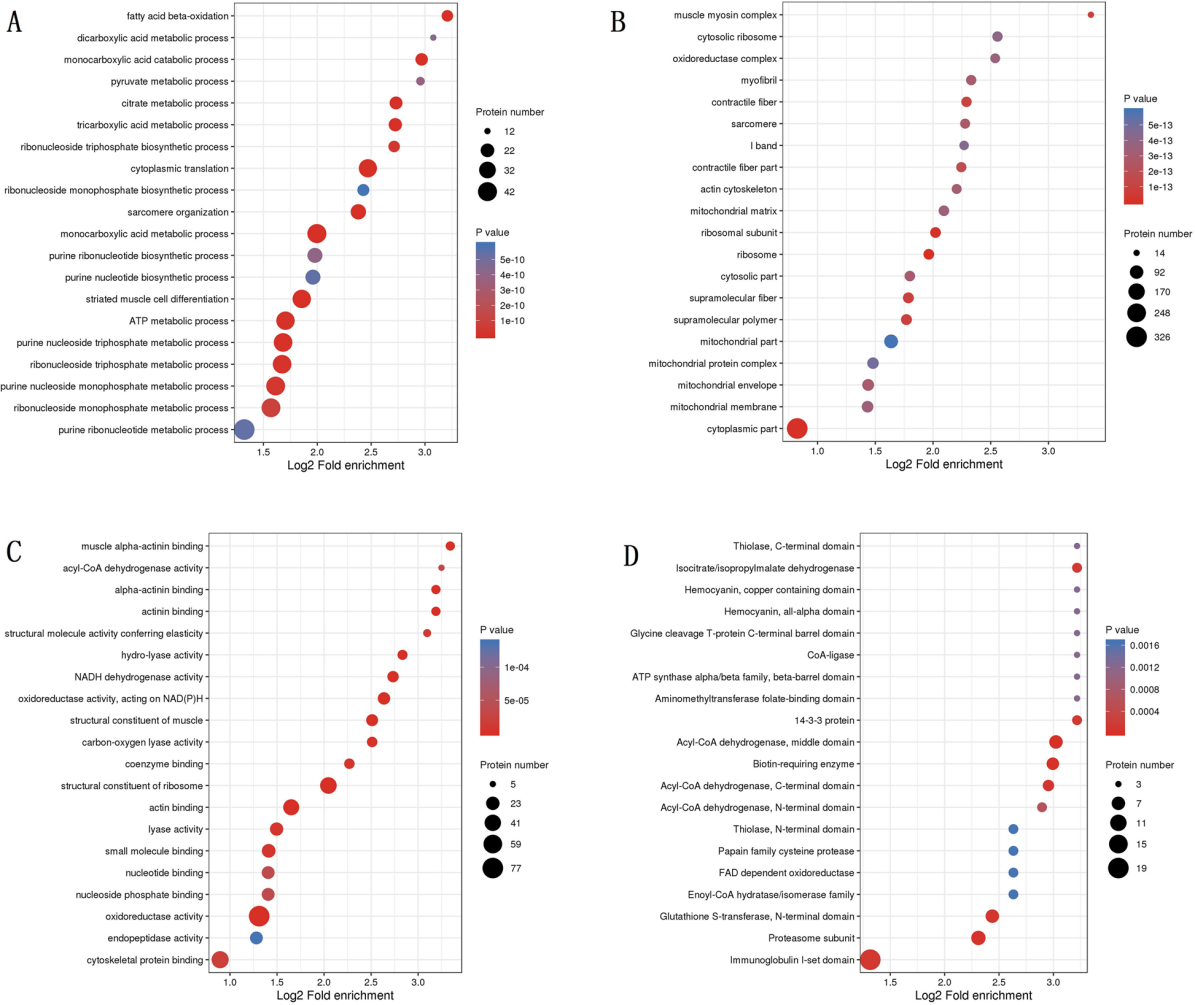
GO and domain enrichment analyses of the identified lysine succinylation proteins in *Solenopsis invicta*. **A** GO enrichment in biological processes. **B** GO enrichment in cellular components. **C** GO enrichment in molecular functions. **D** Protein domain enrichment

The three types of Ksu proteins were elucidated by GO enrichment. As shown in terms of biological process, Ksu proteins were mainly enriched in the monocarboxylic acid metabolic process, fatty acid beta-oxidation, monocarboxylic acid catabolic process, and TCA metabolic process (*p* < 0.0001; Fig. 3A). With respect to cellular component, Ksu proteins were mostly enriched in the cytoplasmic part, ribosomal subunit, ribosome, and muscle myosin complex (*p* < 0.0001; Fig. 3B). In the molecular function, enrichment were observed in Ksu proteins associated with the structural constituents of ribosome, oxidoreductase activity, oxidoreductase activity, acting on NAD(P) H, and actin binding (*p* < 0.0001; Fig. 3C).

Enriched substrates of protein domains were related to the middle domain, C-terminal domain, and N-terminal domain of acyl-CoA dehydrogenase, biotin-requiring enzyme, proteasome subunit, and N-terminal domain of glutathione S-transferase (*p* < 0.0001; Fig. 3D). Furthmore, a few proteins with diverse domains, such as the spectrin repeat, Ca^2+^ insensitive EF hand, EF-hand domain pair, elongation factor Tu domain 2, and elongation factor Tu GTP binding domain, had a series of succinylation sites (S1 Table).

**Fig. 4:**
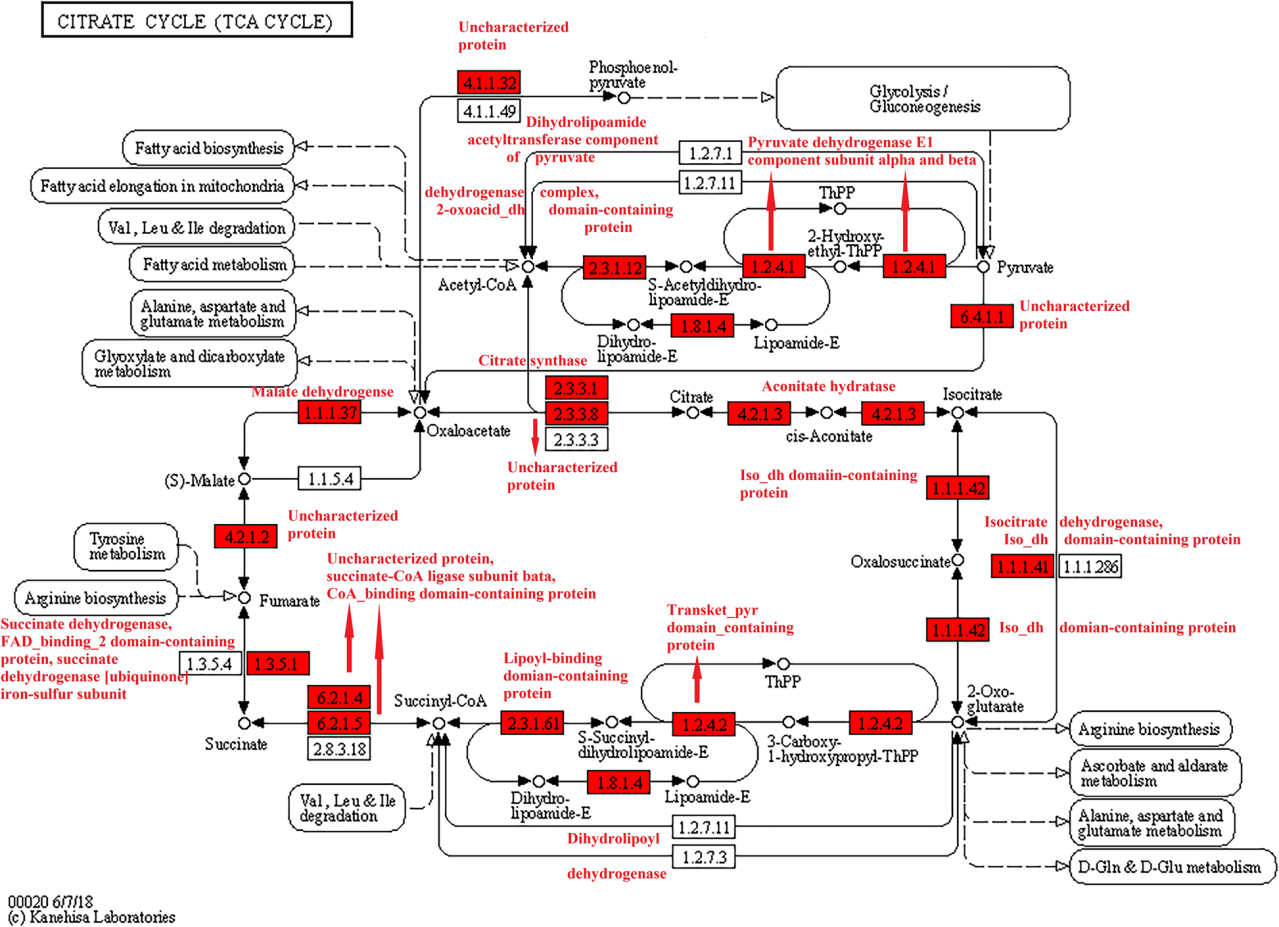
KEGG pathway enrichment in the citrate cycle of *Solenopsis invicta*. Lysine succinylated proteins were labeled with red

**Fig. 5:**
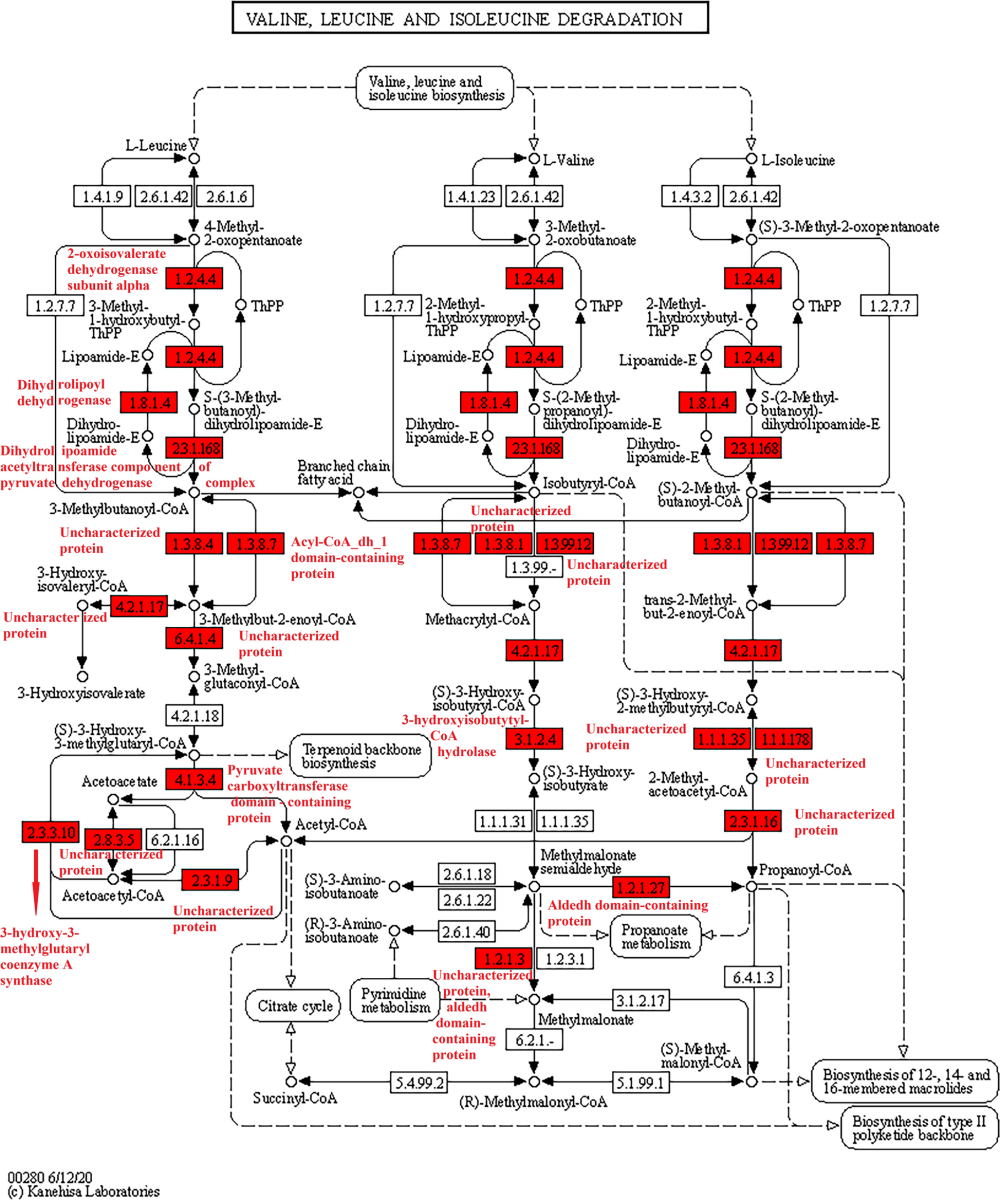
KEGG pathway enrichment in the valine, leucine and isoleucine degradation of *Solenopsis invicta*. The lysine succinylated proteins were labeled with red

**Fig. 6:**
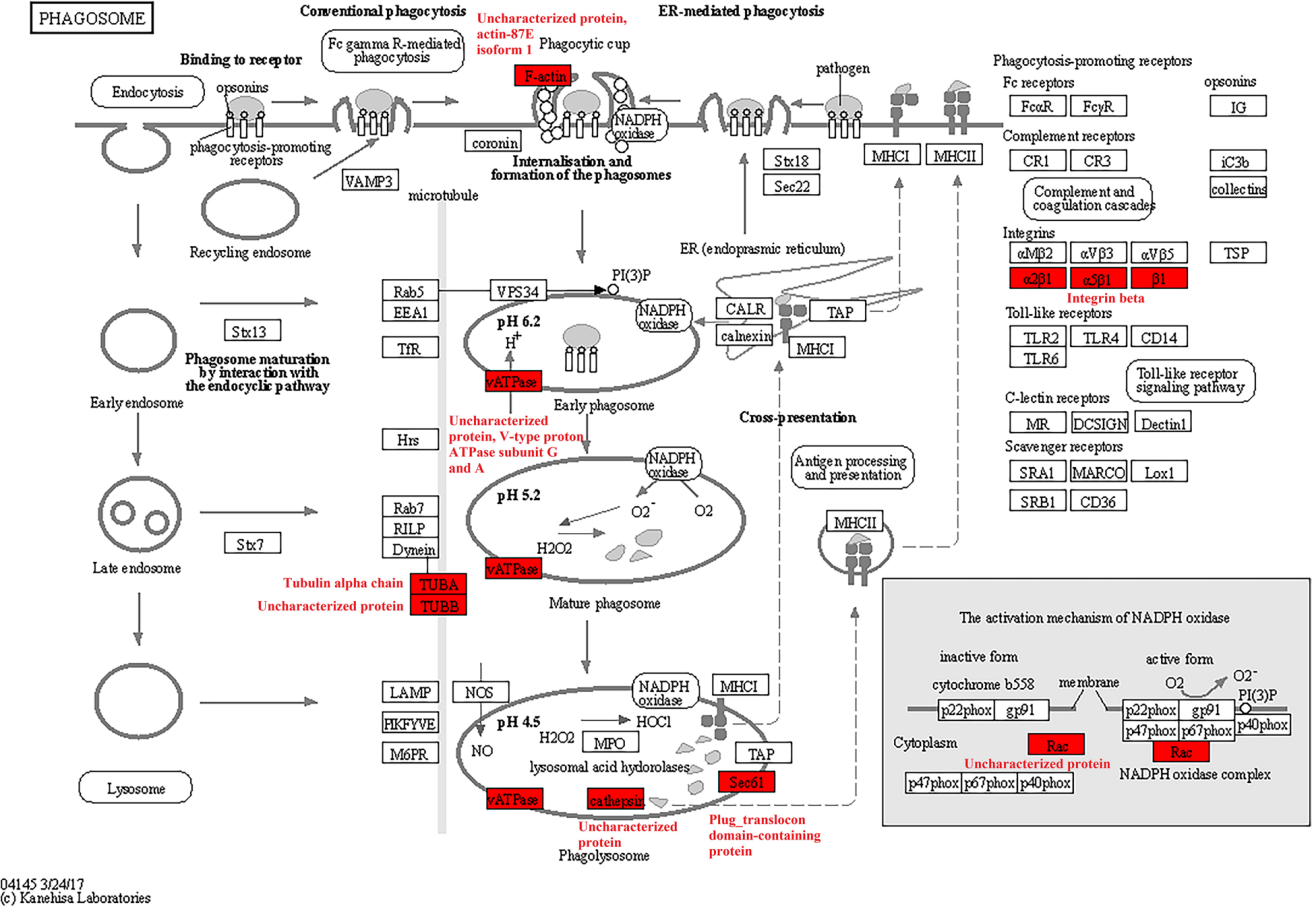
KEGG pathway enrichment in the phagosome of *Solenopsis invicta*. Lysine succinylated proteins were labeled with red

**Fig. 7:**
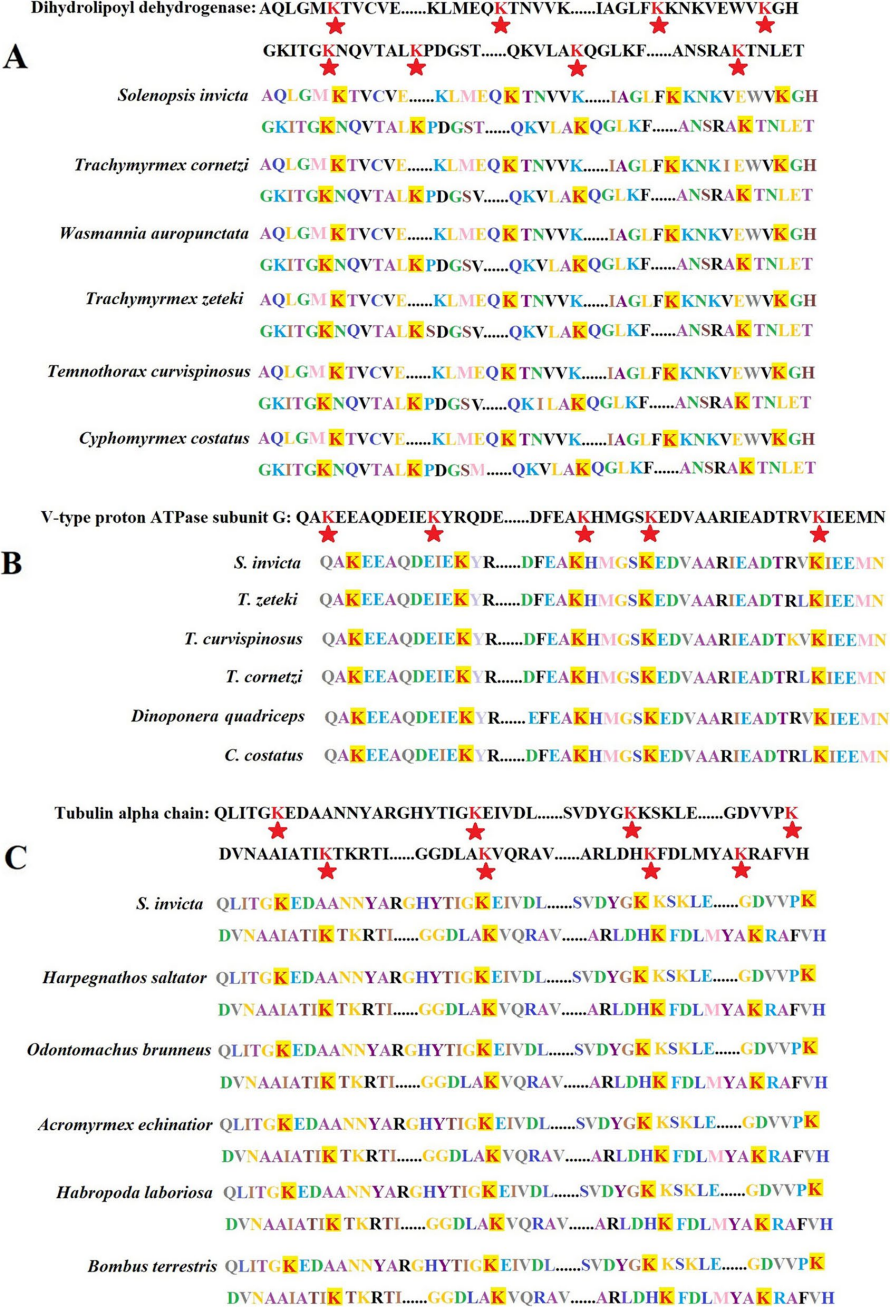
The dihydrolipoyl dehydrogenase, V-type proton ATPase subunit G, and tubulin alpha chain in *Solenopsis invicta* had evolutionary conservatism among diverse ant or bee species. The red stars indicated conserved lysine succinylated residues. **A** Dihydrolipoyl dehydrogenase. **B** V-type proton ATPase subunit G. **C** Tubulin alpha chain

KEGG pathway enrichment analysis found that a total of 25 highly enriched pathways were identified, such as the valine, leucine, and isoleucine degradation, citrate cycle (TCA cycle), ribosome, alanine, aspartate, and glutamate metabolism, propanoate metabolism, pyruvate metabolism, and glycolysis/gluconeogenesis (*p* < 0.0001; S4 Fig.). The TCA cycle included 270 Ksu sites in 26 Ksu proteins, such as the isocitrate dehydrogenase [NAD] subunit, malate dehydrogenase, FAD_binding_2 domain-containing protein, succinate-CoA ligase subunit beta, dihydrolipoyl dehydrogenase, and succinate dehydrogenase (*p* < 0.05; Fig. 4) [16–18]. The valine, leucine, and isoleucine degradation contained 169 Ksu sites in 22 Ksu proteins, such as the dihydrolipoyl dehydrogenase, 3-hydroxy-3-methylglutaryl coenzyme A synthase, pyruvate carboxyltransferase domain-containing protein, aldedh domain-containing protein, 2-oxoisovalerate dehydrogenase subunit alpha, acyl-CoA_dh_1 domain-containing protein, dihydrolipoamide acetyltransferase component of pyruvate dehydrogenase complex, and uncharacterized protein (*p* < 0.05; Fig. 5) [16–18]. There were also a few Ksu proteins in the phagosome, including the V-type proton ATPase subunit G, and tubulin alpha chain (*p* < 0.05; Fig. 6) [16–18]. It was found that the dihydrolipoyl dehydrogenase, which had 18 Ksu sites, and the V-type proton ATPase subunit G, which contained 5 Ksu sites, both had evolutionary conservatism among diverse ant species by sequence alignment analyses (Fig. 7A, B). The residue tubulin alpha chain, which contained 8 Ksu sites, also had evolutionary conservatism among various species of ants and bees (Fig. 7C).

### PPI networks

To study the interactions between different Ksu proteins and their participation in a variety of interaction pathways, PPI analysis was performed with all identified Ksu proteins in *S. invicta*. 203 Ksu proteins were appraised as nodes and interconnected to match PPI networks. 47.29% of the proteins in the networks had more than 50 node degrees, such as uncharacterized protein, ribosomal_L2_C domain-containing protein, ribosomal_L23eN domain-containing protein, and guanine nucleotide-binding protein subunit beta-like protein. 13.79% of the proteins in the PPI networks contained more than 10 Ksu sites, including uncharacterized protein, aconitate hydratase, ATP synthase subunit alpha, transket_pyr domain-containing protein, and dihydrolipoyl dehydrogenase (S2 Table). The dihydrolipoyl dehydrogenase, whose degree was 64, contained 18 sites. The three highly PPI networks included the ribosome, oxidative phosphorylation, and carbohydrate metabolism (S5 Fig. A–C).

### Immunoblotting validation of Ksu proteins in *S. invicta*

**Fig. 8:**
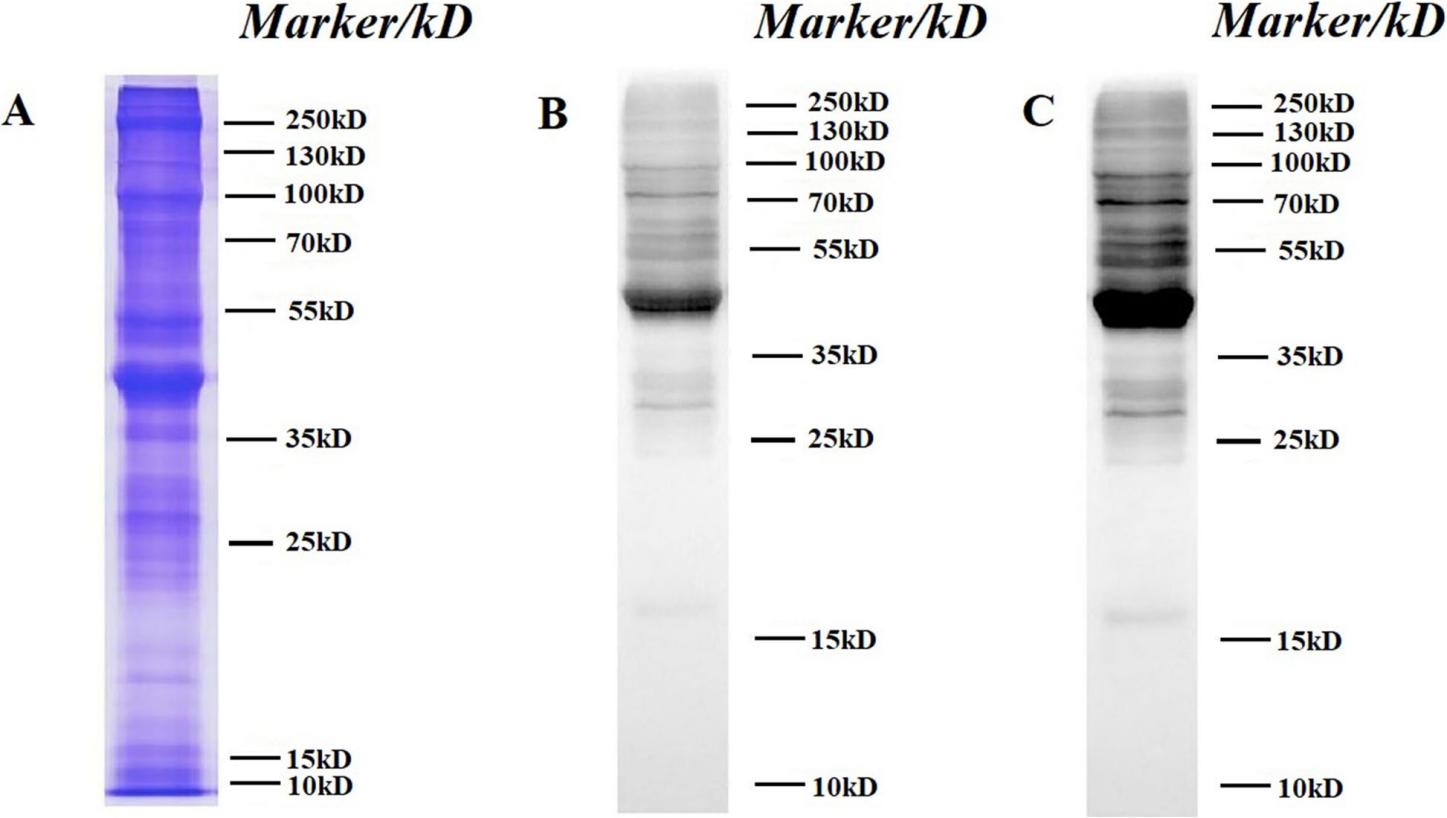
Immunoblotting validation of lysine succinylation (Ksu) proteins in *Solenopsis invicta*. Primary antibody: anti-succinyllysine antibody (PTM-419: Lot: 105032317G009; 1:1000 dilution); second antibody: Thermo, Pierce, horseradish peroxidase-labeled goat anti-mouse IgG antibody, 31,430, 1: 10000 dilution; 20 μg protein/lane. **A** Overall protein level. **B** Short exposure (8 s). **C** Long exposure (15 s)

The distribution of Ksu proteins in *S. invicta* was demonstrated by Western blotting. Figure 8 and S6 Fig. A-C showed the overall protein level and immunoblotting validation of short exposure (8 s) and long exposure (15 s). The result implied that Ksu level in *S. invicta* was quite high. There were many protein bands with a wide range of molecular mass that were investigated.

### Overlap between Ksu and Kac

**Fig. 9:**
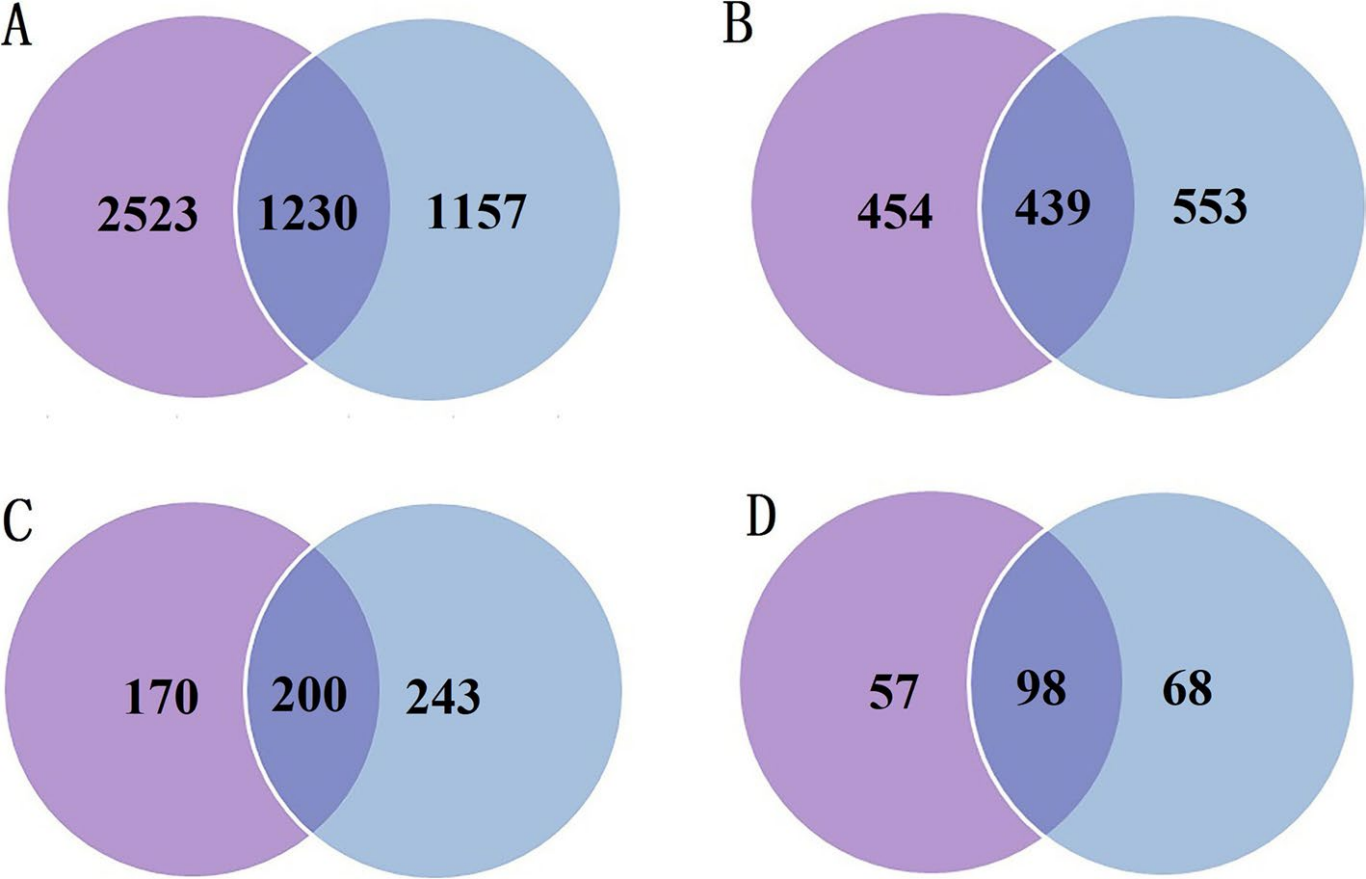
The Venn diagrams of the overlap between succinylation and acetylation in *Solenopsis invicta*. **A** The overlap of all identified sites. **B** The overlap of all identified proteins. **C** The overlap proteins in cytoplasm. **D** The overlap proteins in mitochondria

The sites and proteins in this study of succinylation were compared to those in our previous study of acetylation in *S. invicta* [15]. 1230 sites (32.77%) in 439 proteins (49.16%) were highly overlapped between succinylation and acetylation (Fig. 9A, B). 54.05% of Ksu proteins in cytoplasm were acetylated, while 45.15% of Kac proteins in cytoplasm were succinylated (Fig. 9C). 63.23% of Ksu proteins in mitochondria were acetylated, while 59.03% of Kac proteins in the mitochondria were succinylated (Fig. 9D).

## Discussion

Succinylation proteomic analysis of insects has never been reported before. This was the first time to present the Ksu proteomic analysis and the overlap analysis between succinylation and acetylation in this dangerous invasive species *S*. *invicta*. The results demonstrated that Ksu may play an important role in the allergization, redox metabolism, sugar, fat, and protein metabolism, energy production, immune response, metabolism or biosynthesis of various secondary metabolites, biotic and abiotic stress responses, and nerve signal transduction of *S. invicta*. Furthermore, Ksu and Kac were two ubiquitous protein PTMs participated in a variety of biological processes.

3753 succinylation sites in 893 proteins were tested in our study. Gao et al. [8] implied that *D. rerio* had 552 nonredundant Ksu sites in 264 proteins, which was detected in vertebrates for the first time. 5502 Ksu sites occurring on 2593 proteins were obtained in the *O. sativa* leaves [19]. 1520 Ksu unique sites on 612 proteins were tested in *P. aeruginosa* [11]. Compared with the typical regulation of PTMs (such as phosphorylation), the complexity of succinylation in bacteria seemed to be greater than that in eukaryotes [1]. It was found that the complexity of succinylation was greater in rice than in *S*. *invicta*, while the Ksu complexity was greater in *S*. *invicta* than in bacteria.

In this study, there were enrichments of K_(su)_ xxxxxxK, AK_(su)_, GK_(su)_, K_(su)_ A, and QK_(su)_, which showed different abundances. This indicated that succinyl groups were more likely to modify the proteins with specific amino acid residues. QK was also observed in rice leaf [19], embryo of germinating rice seed [6], and protozoan parasite *Toxoplasma gondii* [20]. K_(su)_ xxxxxxK also appeared in *P. aeruginosa* [11]. These suggested that conserved motifs surrounding Ksu sites were conserved among different species, and alkaline amino acid and hydrophilic amino acids might play pivotal roles in succinylation. The same lysine G was both succinylated and acetylated at the − 1 position in *S. invicta* [15]. This indicated that fatty amino acid was more easily to be modified by both Kac and Ksu. The distribution of amino acids was not biased in the acetylomes of *S. invicta* [15], but there was bias in this study.

The results of GO classification and subcellular localizatioin found that Ksu proteins were involved in a variety of biological processes, and showed a wide subcellular distribution. The Ksu proteins of *D. rerio*, human cell, and mouse liver were predominantly located in the mitochondria [1, 8]. The chloroplast and cytoplasm were the major category of Ksu proteins in the leaves of *P. cablin* [7]. Succinylated proteins were mostly located in cytoplasm in *P. aeruginosa* [11]. Succinate can be formed in the cytoplasm as a residue by passing through the mitochondrial membrane [21]. In this study, cytoplasm was the top preferred cellular component of Ksu proteins in *S. invicta*, followed by mitochondria. The Kac proteins also mainly occurred in the cytoplasm in *S. invicta* [15]. Domain-containing protein of major facilitator superfamily (MFSD) in plasma membrane had only Ksu sites but no Kac sites. Two new putative family members of membrane-bound solute carriers (SLCs), MFSD1 and MFSD3, were identified [22]. The SLCs, which are crucial and contain about 400 transporters, have some physiological functions, for example ion transport, nutrient absorption, and waste scavenging [23]. Both MFSD1 and MFSD3 are conserved in chordates, while MFSD1 is also present in Drosophila [22]. It was found that the venom allergens Sol i II, Sol i III, and Sol i IV, which were located in the extracellular, were succinylated. They were also found to be acetylated in the extracellular in *S. invicta* [15]. There were more sites of venom allergen Sol i II, Sol i III, and Sol i IV (10, 6, and 10 sites, respectively) for succinylation than those for acetylation (7, 4, and 1 sites, respectively). All the 12 Kac sites of venom allergen were overlapped with the Ksu sites. 0.1% of the venom of *S. invicta* consists of four highly allergenic proteins, namely Sol i I, Sol i II, Sol i III, and Sol i IV. These allergenic proteins can lead to anaphylactic shock [24]. These four allergic proteins are different in structure. Sol i IV is unique to *S. invicta* [25]. Soli II and Sol i III are the main protein components of venom, while there are of small amounts of Sol i I and Sol i IV [24]. These demonstrated that both Ksu and Kac may play central roles in the allergization of *S. invicta*. Moreover, there was possible interaction between Ksu and Kac in the modulation of allergization.

The pathways TCA cycle, glycolysis/gluconeogenesis, and pyruvate metabolism, which produced an effect in monitoring sugar, fat, and protein metabolism and energy production for life processes [6], were of significant enrichment. Malate dehydrogenase, which was a key enzyme in TCA cycle, was enriched in the metabolism pathways including cysteine and methionine metabolism, pyruvate metabolism, and glyoxylate and dicarboxylate metabolism. 7 Ksu sites of malate dehydrogenase were overlapped with Kac sites in *S. invicta* [15]. The pathways valine, leucine, and isoleucine degradation, propanoate metabolism, and alanine, aspartate, and glutamate metabolism were also enriched, which suggested that Ksu were related to the metabolism or biosynthesis of various secondary metabolites in *S. invicta*. In the valine, leucine, and isoleucine degradation, all the 19 Kac proteins were overlapped with Ksu ones, and 63 Kac sites were overlapped with Ksu ones in *S. invicta* [15]. Ye and Li [15] showed that in the phagosome there were 7 lysine acetylated proteins with16 Ksu sites, which were also found to be succinylated in our study, including the V-type proton ATPase subunit G, tubulin alpha chain, and actin-87E isoform 1. More proteins, including V-type proton ATPase subunit a, plug_translocon domain-containing protein, integrin beta, and uncharacterized protein, were involved in Ksu than Kac in the phagosome [15]. Ye and Li [15] reported that alpha chain and ATPase subunit G tubulin had evolutionary conservatism in the acetylomes of *S. invicta*. In this study, dihydrolipoyl dehydrogenase located in the mitochondria, tubulin alpha chain located in the cytoskeleton, and ATPase subunit G located in the cytoplasm had evolutionary conservatism among diverse species. The acetylation of tubulin alpha chain and ATPase subunit G in *S. invicta* may participate in the biotic and abiotic stress responses and transduction of nerve signal [15]. Dihydrolipoyl dehydrogenase is a momentous source of reactive oxygen species in mammalian mitochondria [26]. Dihydrolipoyl dehydrogenase was inhibited by heat restriction and participated in *Saccharomyces cerevisiae* aging [27]. The result demonstrated that succinylation may play a role in the redox metabolism of *S. invicta*. Furthermore, the extensive overlap between Ksu and Kac in diverse pathways indicated the possible cooperation between these PTMs in the regulation of sugar, fat, and protein metabolism, energy production, biosynthesis of various secondary metabolites, response to biotic and abiotic stress, transduction of nerve signal, and redox metabolism.

Node degree is a critical parameter to evaluate the significance and relativity of proteins in the network [9]. 47.29% of the Ksu proteins in the network had more than 50 node degrees. The three networks were interlinked by a number of Ksu proteins, suggesting that there were cross-links in the TCA cycle, oxidative phosphorylation, and photosynthesis, and succinylation might adjust these cross-links in *P. cablin* [7]. In our study, the three highly PPI networks were ribosome, oxidative phosphorylation, and carbohydrate metabolism, which indicated that succinylation played a vital role in regulating these biological processes. The first and second largest interaction clusters of Kac proteins were also ribosome, oxidative phosphorylation [15]. These results implied that there were cross-links in the biological process of ribosome and oxidative phosphorylation, and both succinylation and acetylation might monitor these cross-links. In addition, immunoblotting validation showed that there were many Ksu protein bands with a wide range of molecular mass.

Ksu and Kac are two momentous PTMs in proteins, which are participated in the modulation of a variety of biological processes, especially metabolism [28]. 142 proteins were both succinylated and acetylated with 133 overlapping sites in 78 proteins, indicating that Ksu collaboratd or contended with Kac on the same protein [28]. Weinert et al. [1] demonstrated that succinylation extensively overlapped with acetylation in four evolutionarily diverse organisms, increasing the possibility of crosstalk between succinylation and acetylation. There were 27% of human, 56% of *S. cerevisiae*, 57% of mouse, and 66% of *Escherichia coli* Ksu sites, which were acetylated at the same position [1]. In our study, 32.77% of sites in 49.16% of proteins were highly overlapped between succinylation and acetylation. The ratios of *S. cerevisiae* succinylation and acetylation in mitochondrial proteins were similar; the ratios of mitochondrial succinylation were significantly greater than those of mitochondrial acetylation in mouse liver tissue and human cervical cancer cells [1]. In our study, the proportion of Ksu proteins was also greater than that of Kac proteins in mitochondria. The subcellular localizatioins of these PTMs were different in *S. invicta*, mammals, and *S. cerevisiae* [1].

Enriched lysine-succinylated substrates included middle domain, C-terminal domain, and N-terminal domain of acyl-CoA dehydrogenase, biotin-requiring enzyme, proteasome subunit, and N-terminal domain of glutathione S-transferase. Acyl-CoA dehydrogenase, which constitutes a large family of flavoproteins, mostly encodes enzymes of mitochondrial β-oxidation or amino acid metabolism [29, 30]. Acyl-CoA dehydrogenase is a key protein related to lipid metabolism and transport [31]. The pyruvate dehydrogenase complexes, which catalyze the overall conversion of pyruvate to acetyl-CoA and CO_2_, contain three component enzymes: pyruvate decarboxylase, dihydrolipoamide acetyltransferase, and dihydrolipoamide dehydrogenase [6, 32]. The amino acid sequence of dihydrolipoamide acetyltransferase component of pyruvate dehydrogenase complexes, which contained three domain structures, was considerably homologous in human and rat [33]. Over expression of dihydrolipoamide acetyltransferase component may lead to increased immune response [33]. Compared with our previous study, both acyl-CoA dehydrogenase and dihydrolipoamide acetyltransferase component of pyruvate dehydrogenase complexes were succinylated and acetylated in *S. invicta* [15]. The intensive succinylation of the acetyl-CoA metabolism related enzymes indicated that there were complex interactions between acetylation and succinylation [6]. This was further confirmed in our study.

## Conclusion

In conclusion, our research on lysine succinylome may provide basic resources for functional validation of Ksu proteins in this very dangerous invasive pest *S. invicta* and other insects. The details of the cooperation or competition between Ksu and Kac were firstly revealed in *S. invicta*, which may supply rich resources and a starting point for the molecular basic research of regulation on metabolic pathways and other biological processes by the two PTMs. Since there was possible interaction between Ksu and Kac in the modulation of allergization, this provided potential targets for the development of histamines or pesticides using this mechanism against *S. invicta*, which needed further study.

## Materials and methods

### Protein extraction and trypsin digestion

The workers of *S. invicta*, which were gathered from lawn in Guangzhou, China, were mashed into powder by liquid nitrogen. Then, the powder was put into a 5 -mL tube, and lysed in four volumes of buffer containing 1% protease inhibitor cocktail, 8 M urea, 3 μM trichostatin A (TSA), and 50 mM nicotinamide (NAM) on ice. TSA and NAM were inhibitors. Next, the high-intensity ultrasounic processor (Scientz) was used for three times of ultrasonic vibration. The supernatant was collected and the protein concentration was tested by a BCA kit in the light of the manufacturer’s instructions after centrifugation at 4 °C, 12,000 g for 10 min.

1.5 mg protein was digested. The protein solution was reduced with 5 mM dithiothreitol at 56 °C for 30 min and alkylated by adding 11 mM iodoacetamide in the dark at room temperature for 15 min. Next, the dilution of protein sample was carried out by adding 100 mM triethylammonium bicarbonate (TEAB) so that the concentration of urea was < 2 M. Afterwards, the first digestion overnight with trypsin was carried out at a 1: 50 trypsin-to-protein mass ratio, and the second digestion for 4 h with trypsin was conducted at a 1:100 trypsin-to-protein mass ratio.

### Succinylated enrichment

In order to enrich succinylated peptides, the incubation of trypsin peptides lysed in NETN buffer (100 mM NaCl, 50 mM Tris-HCl, 1 mM EDTA, 0.5% NP-40, pH 8.0) was performed with beads of anti-succinyl lysine antibody (PTM Biolabs, Hangzhou, China; PTM-419: Lot: 105032317G009; 1:1000 dilution). After that, the peptides were shaken softly overnight at 4 °C. Then, the binding peptides were eluted from the beads, which were scoured four times with NETN buffer as well as twice with ddH_2_O, with 0.1% trifluoroacetic acid. Afterwards, the peptides were vacuumed.

### LC-MS/MS analysis

The peptides of trypsin were lysed in 0.1% formic acid (FA) (solvent A). Then, the purified peptides were loaded onto a trap column and separated with a reversed-phase analytical column (15-cm length, 75 μm i.d.). The mobile phase components consisted of 0.1% FA in 98% acetonitrile (ACN) (solvent B). The gradient elution conditions were: 6 to 23% solvent B for 26 min, 23 to 35% solvent B for 8 min, 35 to 80% solvent B for 3 min, and 80% solvent B for the last 3 min. All of these were performed on an EASY-nLC 1000 UPLC system at a constant flow rate of 400 nL/min. The peptides were processed by a NSI source and coupled online to the UPLC in Q ExactiveTM Plus (Thermo) by tandem mass spectrometry (MS/MS). The ESD voltage was set to 2.0 kV. The precursor ion scans were ranged from m/z 350 to 1800, and the resolution of complete peptides detected in Orbitrap was 70,000. Then, the normalized collision energy was set to 28 to choose the peptides, and the detection of ion fragments was carried out in the Orbitrap at a resolution of 17,500. A data-dependent process, in which one MS scan was followed by 20 MS/MS scans and 15.0 s dynamic exclusion, was used. MS/MS spectra were generated by accumulating 5E4 ions. The experiments repeated triple.

### Database search

Raw files were handled by MaxQuant (v.1.5.2.8). The generated MS spectra were searched through the database of *S. invicta* protein sequence connected with a reverse decoy database. Trypsin/P was designated as cleavage enzyme and allowed up to four cleavage deletions. The first search mass tolerance of precursor ions was 20 ppm, the main search one was 5 ppm, and the fragment ion one was 0.02 Da. The carbamidomethyl modification on Cys was fixed, while the succinylation and oxidation modification on Met were variable. 40 were the minimal score for Ksu peptides. The false discovery rate (FDR) of identification was set to less than 1%.

### Bioinformatics methods

#### Annotation methods

GO annotation: UniProt-GOA database was used to annotate the Ksu proteome. GO functions of annotated proteins were compared by InterProScan software according to the method of protein sequence alignment if some of the identified proteins were not annotated by UniProt-GOA database. After that, proteins were classified into biological process, cellular component, and molecular function by Gene Ontology annotation.

Domain annotation: InterProScan and InterPro domain database were used to describe the function of the identified protein domains. To determine the potential functions of proteins, the protein sequences were searched according to diagnostic models called signatures at the center of InterPro. InterPro is widely used in extensive analyses of characterization of single protein sequence, whole genomes, and meta-genomes.

KEGG pathway annotation: Kyoto Encyclopedia of Genes and Genomes (KEGG) was applied to annotate and map the Ksu protein pathways. First of all, KEGG database description of annotated proteins was analyzed by KAAS. After that, KEGG mapper was used to map the annotation result to the KEGG pathway database.

Subcellular localization: the subcellular localization was predicted by WOLFPSORT (a subcellular localization predication soft).

#### Motif analysis

Amino acids sequences, which were composed of Ksu sites, were assessed by Motif-x algorithm. The parameters of all sequences of database protein were defaulted. The minimum number of occurrences was set to 20.

#### Functional enrichment

The enrichment of Ksu proteins against all proteins was detected by a two-tailed Fisher’s exact test in the species database for enrichment analyses of GO, KEGG pathway, and protein domains. The GO, pathway, and domains with *p*-values less than 0.05 after correction were considered to be significantly different.

#### Enrichment-based clustering

All the categories and their *p*-values after enrichments were sorted out. Next, those categories, which were concentrated in at least one cluster with *p*-value less than 0.05, were selected. The function x = −log10 (*p*-value) was used to convert the filtered *p*-value matrix. Next, the x score of each functional category was converted to z. Afterwards, one-way hierarchical clustering was used to cluster the z values. The “gplots” R-package was applied to visualize the cluster membership by the heat map of “heatmap.2” function.

#### Protein-protein interaction (PPI) networks

The version 10.5 of STRING database was applied to retrieve the database accessions or sequences of all differentially expressed Ksu protein for PPIs. The interactions among the Ksu proteins were chosen in the dataset, while external candidates were excluded. The interaction of confidence score > 0.7 (high confidence) was presented. The STRING database was used to obtain PPI networks, while R package “networkD3” was applied to visualize the networks.

### Immunoblotting validation

Proteins were separated by 12% SDS-PAGE. Then, the separated proteins were diverted to an NC membrane (BioRad, 0.2 μm). After being blocked with 5% bovine serum albumin for 90 min, the membrane was incubated with a 1:1000 dilution of pan anti-succinyl lysine antibody (PTM Biolabs, Hangzhou, China). Subsequently, Ksu proteins were revealed. The second antibody was 1:10,000 diluted with horseradish peroxidase-labeled goat anti-mouse IgG antibody.

## Supplementary Information

**Additional file 1: S1 Fig.** Length distribution of lysine succinylation (Ksu) peptides in *Solenopsis invicta*. (A) Experiment I. (B) Experiment II. (C) Experiment III. **S2 Fig.** Amounts of succinylated sites per protein. (A) Experiment I. (B) Experiment II. (C) Experiment III. **S3 Fig.** MS^2^ spectra of lysine succinylated sequence. (A) The amino acid position K29 in Sol i II. (B) K40 in Sol i II. (C) K71 in Sol i III. (D) K204 in Sol i III. (E) K74 in Sol i IV. (F) K91 in Sol i IV. **S4 Fig.** KEGG pathway enrichment analyses of the identified lysine succinylation proteins in *Solenopsis invicta*. **S5 Fig.** Protein-protein interaction (PPI) networks analyses of succinylated proteins in *Solenopsis invicta*. (A) Ribosome. (B) Oxidative phosphorylation. (C) Carbohydrate metabolism. **S6 Fig.** Immunoblotting validation of lysine succinylation (Ksu) proteins in *Solenopsis invicta*. Primary antibody: anti-succinyllysine antibody (PTM-419: Lot: 105032317G009; 1:1000 dilution); second antibody: Thermo, Pierce, horseradish peroxidase-labeled goat anti-mouse IgG antibody, 31,430, 1: 10000 dilution; 20 μg protein/lane. Lane 2, 3, 5, and 6 were not related to this experiment. (A) Overall protein level. Lane 4 in red box corresponded to Fig. 8A. Lane 1 was experimental repetition. (B) Short exposure (8 s). Lane 1 in red box corresponded to Fig. 8B. Lane 4 was experimental repetition. (C) Long exposure (15 s). Lane 1 in red box corresponded to Fig. 8C. Lane 4 was experimental repetition.

**Additional file 2: S1 Table.** Lysine succinylated proteins with various domains in *Solenopsis invicta*.

**Additional file 3: S2 Table.** Lysine succinylated proteins containing diverse degrees of protein–protein interaction in *Solenopsis invicta*.

## Data Availability

The datasets generated and/or analysed during the current study are available in the Mendeley Data repository, [PERSISTENT WEB LINK TO DATASETS 10.17632/pj5ycyxdzx.1].

## References

[1] Weinert BT, Scholz C, Wagner SA, Iesmantavicius V, Su D, Daniel JA, Choudhary C (2013). Lysine succinylation is a frequently occurring modification in prokaryotes and eukaryotes and extensively overlaps with acetylation. Cell Rep.

[2] Smestad J, Erber L, Chen Y, Maher LJ (2018). Chromatin succinylation correlates with active gene expression and is perturbed by defective TCA cycle metabolism. Science.

[3] Wagner GR, Bhatt DP, O’Connell TM, Thompson JW, Dubois LG, Backos DS, Yang H, Mitchell GA, Ilkayeva OR, Stevens RD (2017). A class of reactive acyl-CoA species reveals the non-enzymatic origins of protein acylation. Cell Metab.

[4] Zhang Z, Tan M, Xie Z, Dai L, Chen Y, Zhao Y (2011). Identification of lysine succinylation as a new post-translational modification. Nat Chem Biol.

[5] Park J, Chen Y, Tishkoff DX, Peng C, Tan M, Dai L, Xie Z, Zhang Y, Zwaans BM, Skinner ME (2013). SIRT5-mediated lysine desuccinylation impacts diverse metabolic pathways. Mol Cell.

[6] He D, Wang Q, Li M, Damaris RN, Yi X, Cheng Z, et al. Global proteome analyses of lysine acetylation and succinylation reveal the widespread involvement of both modification in metabolism in the embryo of germinating rice seed. J Proteome Res. 2016:879–90. 10.1021/acs.jproteome.5b00805. 10.1021/acs.jproteome.5b00805 26767346

[7] Wang XB, Chen XZ, Li JR, Zhou XX, Liu YT, Zhong LT, Tang Y, Zheng H, Liu JY, Zhan RT, Chen LK (2019). Global analysis of lysine succinylation in patchouli plant leaves. Hortic Res.

[8] Gao Y, Lee H, Kwon OK, Tan M, Kim K, Lee S (2019). Global proteomic analysis of lysine succinylation in zebrafish (*Danio rerio*). J Proteome Res.

[9] Wang J, Li L, Chai R, Zhang Z, Qiu H, Mao X, Hao Z, Wang Y, Sun G (2019). Succinyl-proteome profiling of *Pyricularia oryzae*, a devastating phytopathogenic fungus that causes rice blast disease. Sci Rep.

[10] Zhen S, Deng X, Wang J, Zhu G, Cao H, Yuan L (2016). First comprehensive proteome analyses of lysine acetylation and succinylation in seedling leaves of *Brachypodium distachyon* L. Sci Rep.

[11] Gaviard C, Broutin I, Cosette P, De E, Hardouin J (2018). Lysine succinylation and acetylation in *Pseudomonas aeruginosa*. J Proteome Res.

[12] Cheng D, Chen S, Huang Y, Pierce NE, Riegler M, Yang F, Huang Y, Pierce NE, Riegler M, Yang F, Zeng L, Lu YY, Liang GW, Xu YJ (2019). Symbiotic microbiota may reflect host adaptation by resident to invasive ant species. PLoS Pathog.

[13] DeShazo RD, Banks WA (1994). Medical consequences of multiple fire ant stings occur-ring indoors. J Allergy Clin Immunol.

[14] Ross KG (1994). Exotic ants—biology, impact, and control of introduced species. Science.

[15] Ye JW, Li J (2020). First proteomic analysis of the role of lysine acetylation in extensive functions in *Solenopsis invicta*. PLoS One.

[16] Kanehisa M, Goto S (2000). KEGG: Kyoto encyclopedia of genes and genomes. Nucleic Acids Res.

[17] Kanehisa M (2019). Toward understanding the origin and evolution of cellular organisms. Protein Sci.

[18] Kanehisa M, Furumichi M, Sato Y, Ishiguro-Watanabe M, Tanabe M (2021). KEGG: integrating viruses and cellular organisms. Nucleic Acids Res.

[19] Zhou H, Finkemeier I, Guan WX, Tossounian MA, Wei B, Yong D, et al. Oxidative stress-triggered interactions between the succinyl- and acetyl-roteomes of rice leaves. Plant Cell Environ. 2017. 10.1111/pce.13100. 10.1111/pce.13100 29126343

[20] Li XL, Hu X, Wan YJ, Xie GZ, Li XZ, Chen D, Cheng ZY, Yi XL, Liang SH, Tan F (2014). Systematic identification of the lysine succinylation in the protozoan parasite *toxoplasma gondii*. J Proteome Res.

[21] Hausinger RP (2004). FeII/alpha-ketoglutarate-dependent hydroxylases and related enzymes. Crit Rev Biochem Mol Biol.

[22] Perland E, Hellsten SV, Lekholm E, Eriksson MM, Arapi V, Fredriksson R (2017). The novel membrane-bound proteins MFSD1 and MFSD 3 are putative SLC transporters affected by altered nutrient intake. J Mol Neurosci.

[23] Hediger MA, Romero MF, Peng JB, Rolfs A, Takanaga H, Bruford EA (2004). The ABCs of solute carriers: physiological, pathological and therapeutic implications of human membrane transport proteins. Pflugers Arch - Eur J Physiol.

[24] Hoffman DR (1993). Allergens in Hymenoptera venom: XXIV. The amino acid sequences of imported fire ant venom allergens sol i II, sol i III, and sol i IV. J Allergy Clin Immunol.

[25] Nugent JS, More DR, Hagan LL, Demain JG, Whisman BA, Freeman TM (2004). Cross-reactivity between allergens in the venom of the common striped scorpi on and the imported fire ant. J Allergy Clin Immunol.

[26] Starkov AA, Fiskum G, Chinopoulos C, Lorenzo BJ, Browne SE, Patel MS, Beal MF (2004). Mitochondrial alpha-ketoglutarate dehydrogenase complex generates reactive oxygen species. J Neurosci.

[27] Tahara EB, Barros MH, Oliveira GA, Netto LES, Kowaltowski AJ (2007). Dihydrolipoyl dehydrogenase as a source of reactive oxygen species inhibited by caloric restriction and involved in *Saccharomyces cerevisiae* aging. FASEB J.

[28] Choudhary C, Kumar C, Gnad F, Nielsen ML, Rehman M, Walther TC, Olsen JV, Mann M (2009). Lysine acetylation targets protein complexes and co-regulates major cellular functions. Science.

[29] Dschner K, Thalheim C, Guha C, Brennicke A, Binder S (1999). In plants a putative isovaleryl-CoA-dehydrogenase is located in mitochondria. Plant Mol Biol.

[30] Ghisla S, Thorpe C (2010). Acyl-CoA dehydrogenases. FEBS J.

[31] Wheeler DE, Tuchinskaya II, Buck NA, Tabashnik BE (2000). Hexameric storage proteins during metamorphosis, egg production in the diamondback moth, *Plutella xylostella* (Lepidoptera). J Insect Physiol.

[32] Turner SL, Russell GC, Williamson MP, Guest JR (1993). Restructuring an interdomain linker in the dihydrolipoamide acetyltransferase component of the pyruvate dehydrogenase complex of *Escherichia coli*. Protein Eng Des Sel.

[33] Wang L, Kaneko S, Kagaya M, Ohno H, Honda M, Kobayashi K (2002). Molecular cloning, and characterization and expression of dihydrolipoamide acetyltransferase component of murine pyruvate dehydrogenase complex in bile duct cancer cells. J Gastroenterol.

