# Supplementary material for: First analyses of lysine succinylation proteome and overlap between succinylation and acetylation in Solenopsis invicta Buren (Hymenoptera: Formicidae)
Source: BMC Genomics. 2022 Jan 17;23:61. doi: 10.1186/s12864-021-08285-8 (PMC8764763; doi:10.1186/s12864-021-08285-8)
Supplement: Supplementary file 1 — Additional file 1: S1 Fig. Length distribution of lysine succinylation (Ksu) peptides in Solenopsis invicta. (A) Experiment I. (B) Experiment II. (C) Experiment III. S2 Fig. Amounts of succinylated sites per protein. (A) Experiment I. (B) Experiment II. (C) Experiment III. S3 Fig. MS2 spectra of lysine succinylated sequence. (A) The amino acid position K29 in Sol i II. (B) K40 in Sol i II. (C) K71 in Sol i III. (D) K204 in Sol i III. (E) K74 in Sol i IV. (F) K91 in Sol i IV. S4 Fig. KEGG pathway enrichment analyses of the identified lysine succinylation proteins in Solenopsis invicta. S5 Fig. Protein-protein interaction (PPI) networks analyses of succinylated proteins in Solenopsis invicta. (A) Ribosome. (B) Oxidative phosphorylation. (C) Carbohydrate metabolism. S6 Fig. Immunoblotting validation of lysine succinylation (Ksu) proteins in Solenopsis invicta. Primary antibody: anti-succinyllysine antibody (PTM-419: Lot: 105032317G009; 1:1000 dilution); second antibody: Thermo, Pierce, horseradish peroxidase-labeled goat anti-mouse IgG antibody, 31,430, 1: 10000 dilution; 20 μg protein/lane. Lane 2, 3, 5, and 6 were not related to this experiment. (A) Overall protein level. Lane 4 in red box corresponded to Fig. 8A. Lane 1 was experimental repetition. (B) Short exposure (8 s). Lane 1 in red box corresponded to Fig. 8B. Lane 4 was experimental repetition. (C) Long exposure (15 s). Lane 1 in red box corresponded to Fig. 8C. Lane 4 was experimental repetition. [file 12864_2021_8285_MOESM1_ESM.pdf]

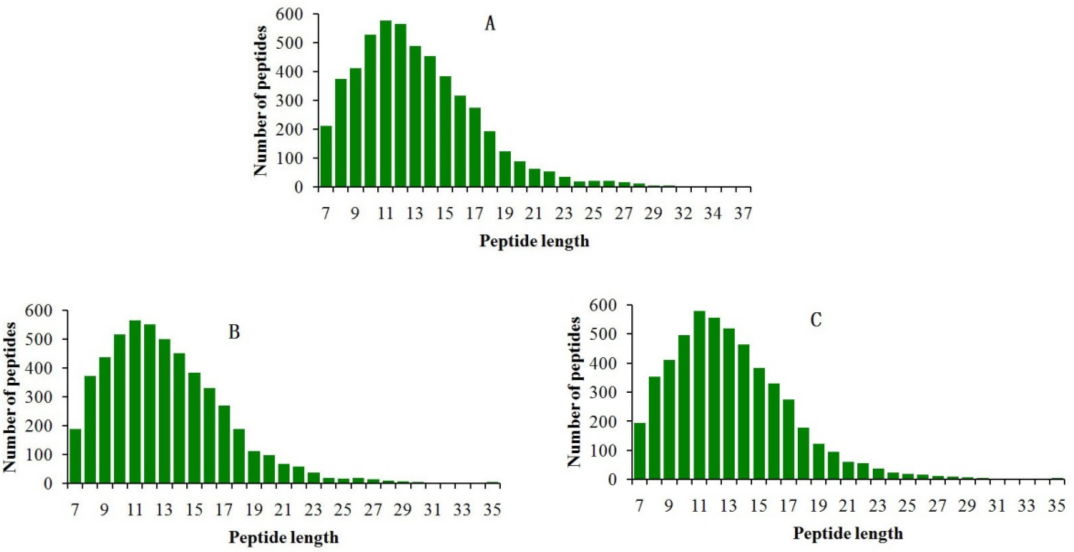

S1 Fig. Length distribution of lysine succinylation (Ksu) peptides in *Solenopsis invicta*. (A) Experiment I. (B) Experiment II. (C) Experiment III.

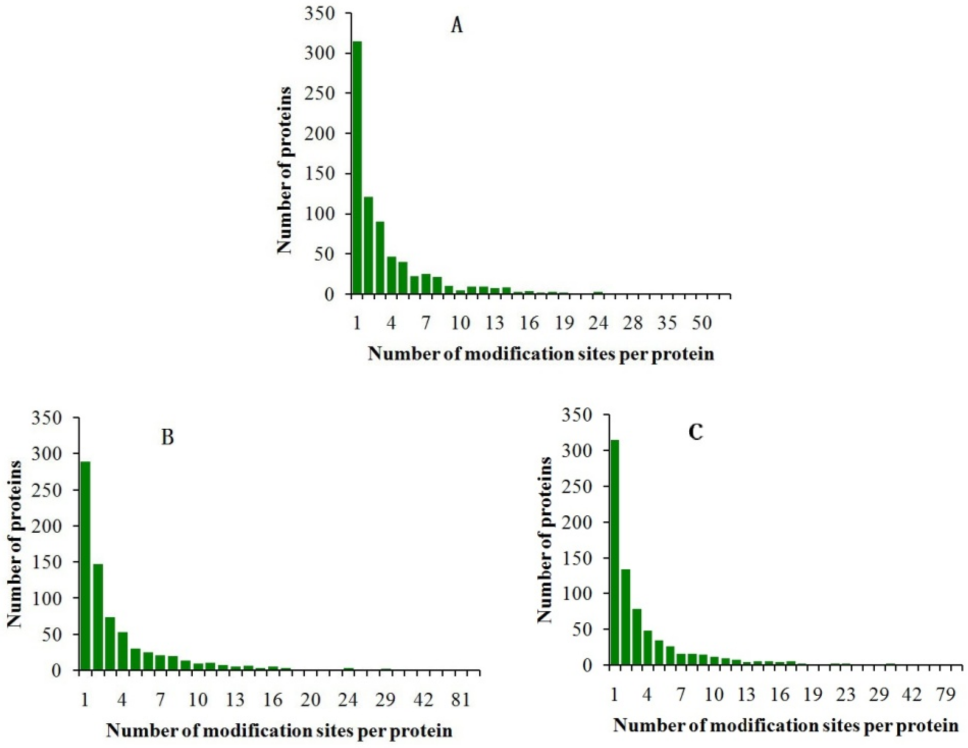

S2 Fig. Amounts of succinylated sites per protein. (A) Experiment I. (B) Experiment II. (C) Experiment III.

A

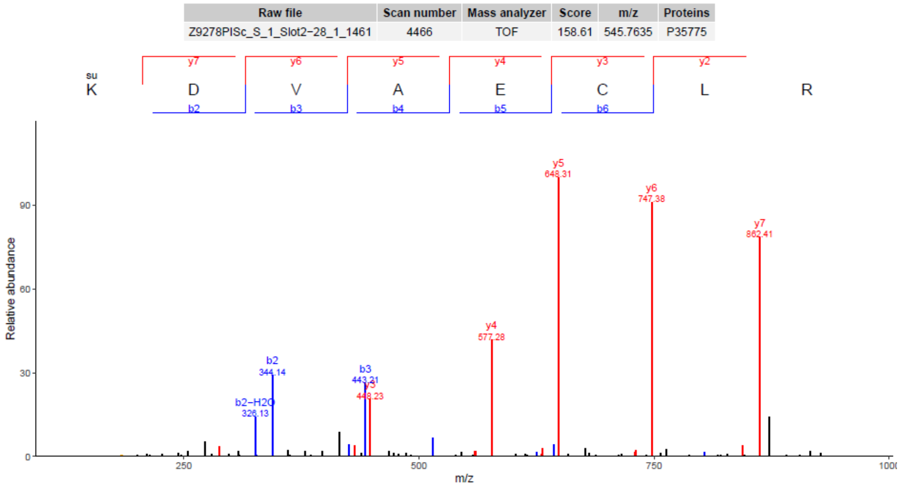

Raw file | Scan number | Mass analyzer | Score | m/z | Proteins
Z9278PISc_S_1_Slot2-28_1_1461 | 4466 | TOF | 158.61 | 545.7635 | P35775
su K D V A E C L R
y7 y6 y5 y4 y3 y2
b2 b3 b4 b5 b6
Relative abundance
m/z

B

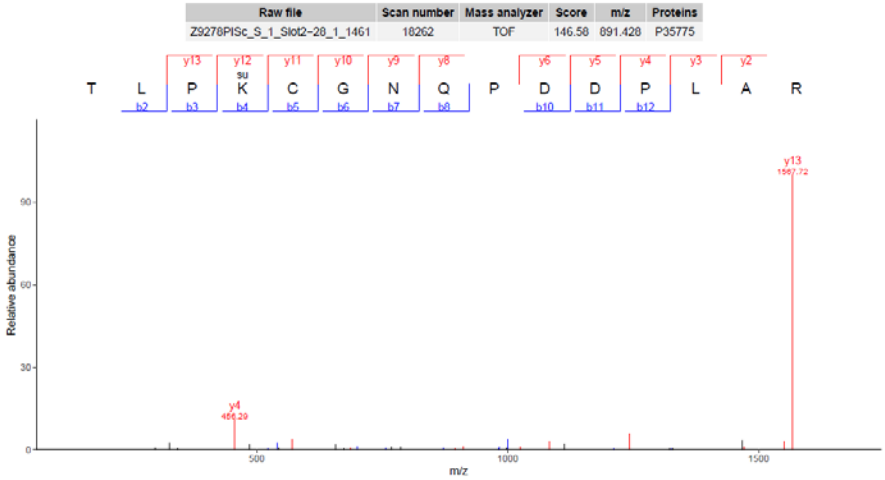

Raw file | Scan number | Mass analyzer | Score | m/z | Proteins
Z9278PISc_S_1_Slot2-28_1_1461 | 18262 | TOF | 146.58 | 891.428 | P35775
T L P su K C G N Q P D D P L A R
y13 y12 y11 y10 y9 y8 y6 y5 y4 y3 y2
b2 b3 b4 b5 b6 b7 b8 b10 b11 b12
Relative abundance
m/z

C

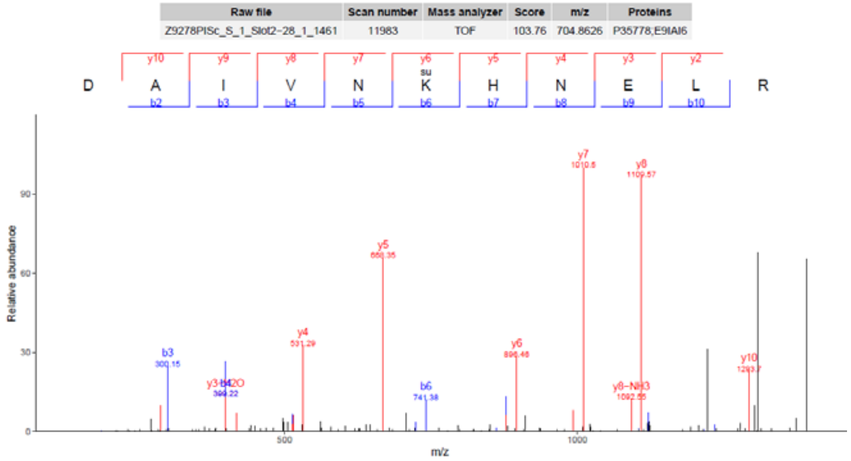

Raw file | Scan number | Mass analyzer | Score | m/z | Proteins
Z9278PISc_S_1_Slot2-28_1_1461 | 11983 | TOF | 103.76 | 704.8626 | P35778;E9IAI6
D A I V N su K H N E L R
y10 y9 y8 y7 y6 y5 y4 y3 y2
b2 b3 b4 b5 b6 b7 b8 b9 b10
Relative abundance
m/z

D

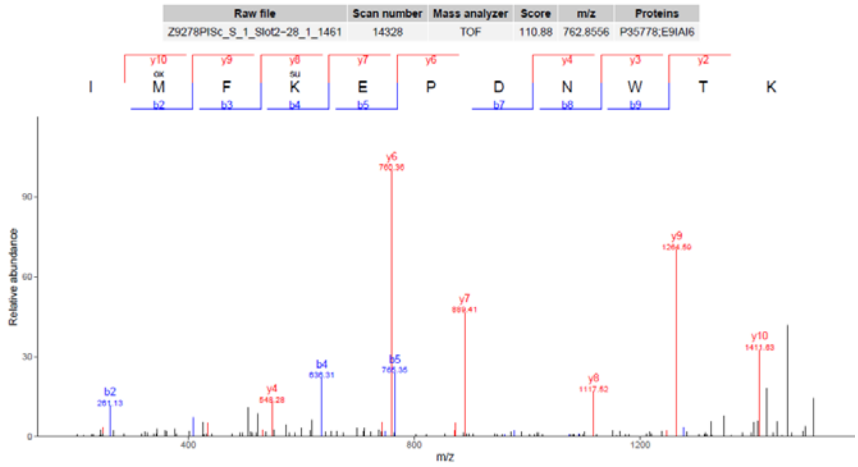

E

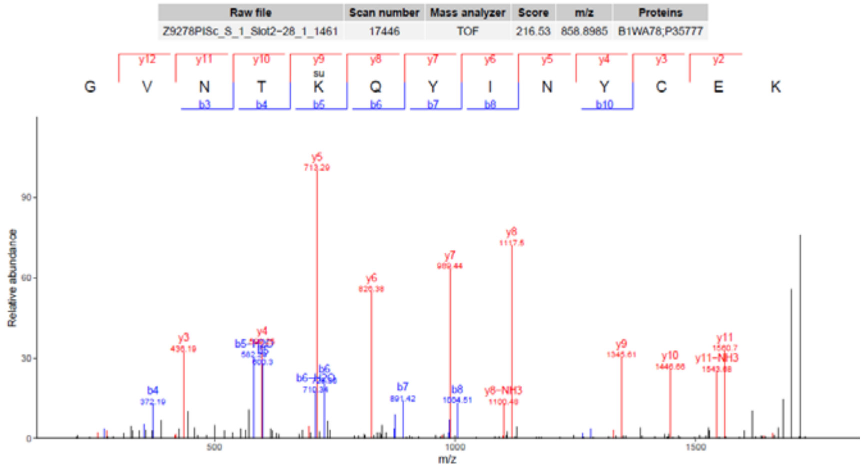

F

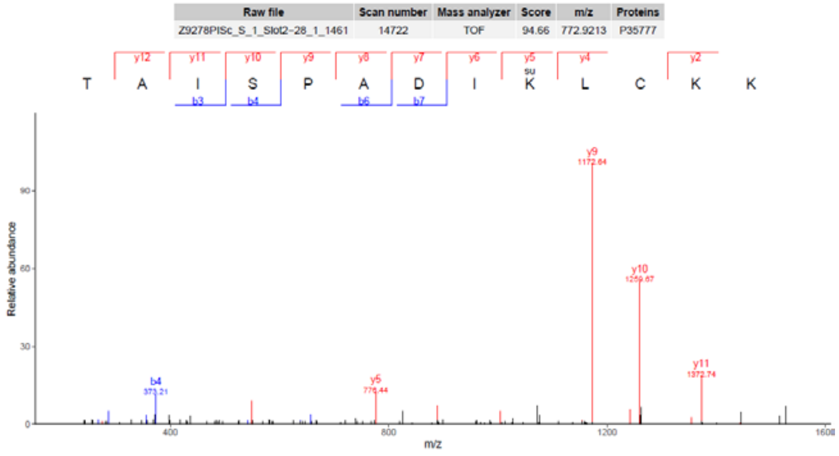

S3 Fig. MS^2 spectra of lysine succinylated sequence. (A) The amino acid position K29 in Sol i II. (B) K40 in Sol i II. (C) K71 in Sol i III. (D) K204 in Sol i III. (E) K74 in Sol i IV. (F) K91 in Sol i IV.

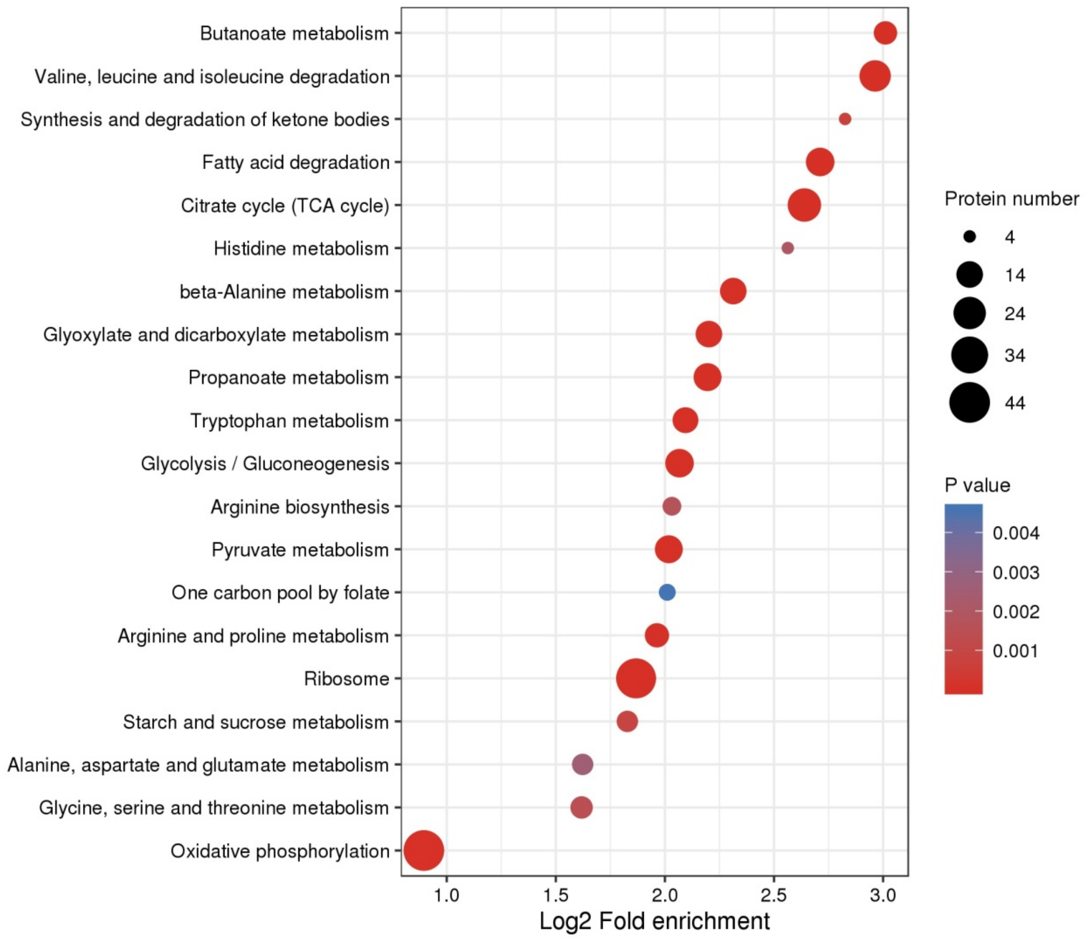

S4 Fig. KEGG pathway enrichment analyses of the identified lysine succinylation proteins in *Solenopsis invicta*.

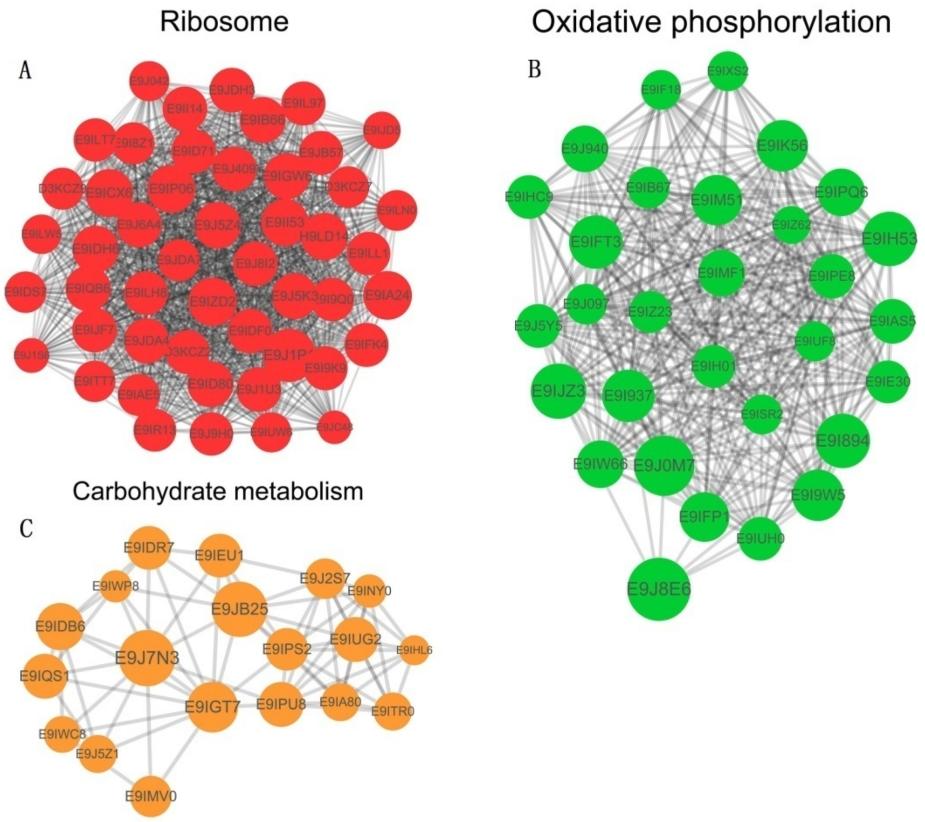

S5 Fig. **Protein-protein interaction (PPI) networks analyses of succinylated proteins in *Solenopsis invicta*.** (A) Ribosome. (B) Oxidative phosphorylation. (C) Carbohydrate metabolism.

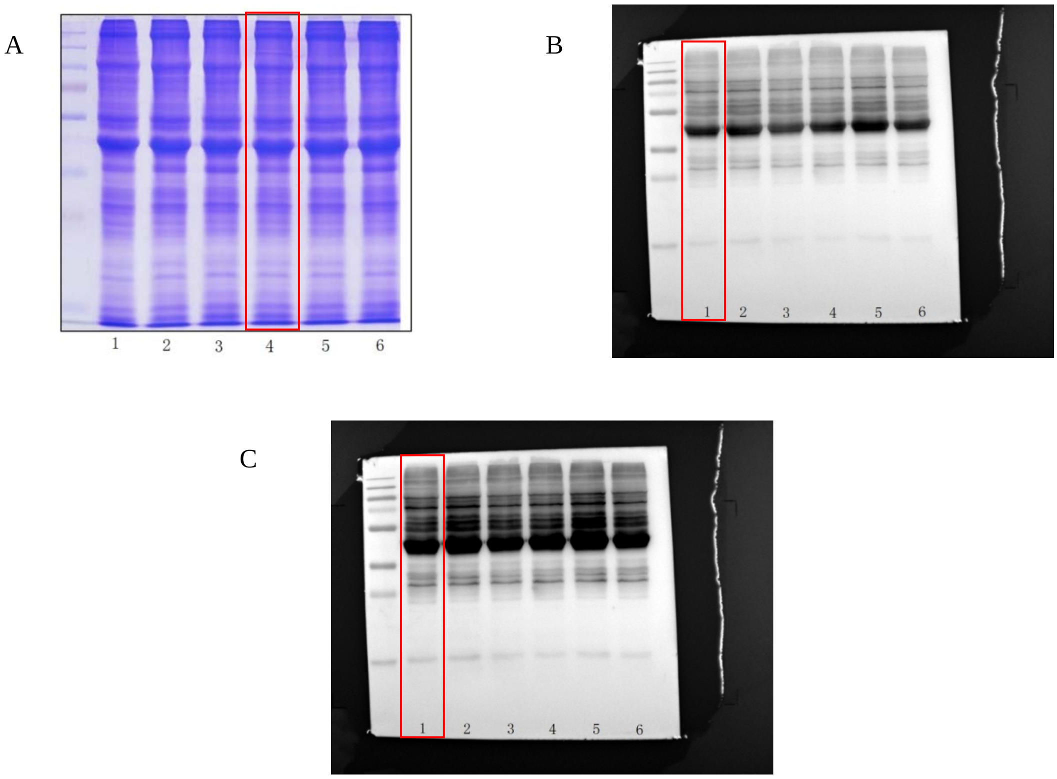

S6 Fig. Immunoblotting validation of lysine succinylation (Ksu) proteins in *Solenopsis invicta*. Primary antibody: anti-succinyllysine antibody (PTM-419: Lot: 105032317G009; 1:1000 dilution); second antibody: Thermo, Pierce, horseradish peroxidase-labeled goat anti-mouse IgG antibody, 31430, 1: 10000 dilution; 20 μg protein/lane. Lane 2, 3, 5, and 6 were not related to this experiment. (A) Overall protein level. Lane 4 in red box corresponded to Figure 8A. Lane 1 was experimental repetition. (B) Short exposure (8s). Lane 1 in red box corresponded to Figure 8B. Lane 4 was experimental repetition. (C) Long exposure (15s). Lane 1 in red box corresponded to Figure 8C. Lane 4 was experimental repetition.
